# NEWS

**DOI:** 10.7189/jogh.04.020203

**Published:** 2014-12

## DEMOGRAPHY

➢ It is widely agreed that 2.1 children per woman is the replacement rate for stable populations in developed countries, and slightly higher in developing countries. Increasingly, fertility rates have fallen below this; 1.5 in China, 1.6 in Europe, 1.4 in Japan and 1.3 in South Korea, and are also falling in several less wealthy south Asian countries. Soon, half the world’s population will live in countries where the population is not reproducing itself. Fewer children means fewer workers to support a growing number of pensioners, leading to efforts to boost fertility eg, tax incentives, child benefits and child–care provision, with mixed results. However, some experts believe that this is an over–estimate, as higher educational levels means that populations are independent for longer. This leads to estimated optimal fertility rates of 1.5–1.8; and countries with high immigrant rates need lower replacement rates. (*The Economist*, 31 May 2014)

➢ A UN population report shows that the global urban population is set to rise to more than 6 billion people by 2050. Africa and Asia will face challenges in meeting the needs of their growing urban populations, from basic services – such as education – to energy, infrastructure and employment. Africa is predicted to experience the highest rate of urbanisation, and there is an anticipated 40 megacities worldwide, each with populations of at least 10 million. Delhi, Shanghai and Tokyo will be the world’s most populous cities. The global rural population will decline as urbanisation increases, and the UN says that cities must generate income and employment and invest in the necessary infrastructure to support their citizens and protect the environment. (*The Guardian*, 10 Jul 2014)

➢ According to an IPSOS survey, 77% of people in developed countries are happy, although most wish their lives were simpler. Whilst most were happy with their personal lives, there was concern about the future, globalisation and the effects of inequality. The survey questioned 16 000 people on 10 areas, including the digital revolution, health care, generational tensions and immigration. It found that people in developing countries felt both more positive about globalisation compared to developed countries, and more pressure to make money and display their success. (*The Guardian*, 16 Jul 2014)

➢ According to Myanmar’s Central Census Commission, the country’s population is probably less than the accepted 60 million people. The country has lacked reliable population estimates for 30 years. The first census for 30 years controversially asked people to identify their ethnicity and religion, causing tensions in the multi–ethnic country. One group was excluded as it refused to self–identify using the government’s preferred term, and census–takers could not gain access to other rebel–controlled areas. (*Irrawaddy Magazine*, 14 Aug 2014)

➢ The upper range of the latest UN population projections suggest that the world’s population could increase to 12 billion by 2011, from its current 7.2 billion. These trends have been apparent for some time, with global population growth slowing down and stabilising, although not stopping. Long–range population forecasts can be unreliable, as the assumptions made about birth rates are often off–target, eg, the fall in Iran and Bangladesh’s fertility rates from 6 to 2 from 1980–2014 was not foreseen. Therefore, if Africa’s birth rates, which form much of the projected increase, experience the same decline, then the 2100 population figures could be very different. (*The Economist*, 24 Sep 2014)

## ECONOMY

➢ To mark the 50th anniversary of the founding of the Group of 77 (G77) developing countries, the group published a re–affirmation of the needs of developing countries, noting that it was established in 1964 to address imbalances in the global economy which are still prevalent in 2014. It emphasised the importance of World Trade Organization agreements on Trade–Related Aspects of Intellectual Property Rights for public health and access to medicines, and called on developed countries not to take action, eg, trade measures, against developing countries making use of these flexibilities. It also called for more technology transfers, capacity–building, wider South–South co–operation, financial reform, and for the “democratic deficit” in global economic governance to be addressed. (*IP–Watch*, 18 Jun 2014)

➢ The “BRICS” countries of Brazil, Russia, India, China and South Africa have reached a broad agreement on their US$ 100 billion development bank, and are expected to sign a treaty to officially launch the bank in July 2014. The new bank symbolises the growing influence of emerging economies in the global financial architecture. Although consensus on some technical aspects of the bank has still to be reached, it is anticipated that it will help fund the growing demand for project funding which is not completely met by global agencies. (*Reuters*, 7 Jul 2014)

➢ According to analysis from the World Bank Group, Ebola’s economic impact could increase eight–fold in already–fragile Guinea, Liberia and Sierra Leone – a potentially catastrophic blow. This could be limited if the epidemic is contained. In the short–term, this could reduce economic growth in Guinea by 2.1%, 3.4% in Liberia and 3.3% in Sierra Leone – equivalent to losses of US$ 359 million. If Ebola is not contained, the estimated economic damage increases to US$ 809 million. Inflation and food prices are already rising due to shortages, panic buying and speculation. In line with other epidemics, most economic damage arises from aversion behaviour rather than direct costs, and underlines the need for a concerted international response, including humanitarian aid, fiscal support, screening facilities and strengthening health systems. (*World Bank*, 17 Sep 2014)

➢ Jean Tirole, an economics professor at the University of Toulouse, France, won the 2014 Nobel Prize for economics. His work on understanding and regulating industries with a few powerful firms is becoming increasingly important as public monopolies such as water, electricity and telecoms are privatised, and has been adopted by competition regulators worldwide. Upon announcing the winner, Staffan Normark said “this year’s prize in economic sciences is about taming powerful firms”. (*The Guardian*, 13 Oct 2014)

➢ According to the Credit Suisse global wealth report, the richest 1% of the world’s population are getting wealthier, have net worth of about US$ 800 000 or more, and own more than 48% of global wealth – and the bottom 50% own less than 1% of global wealth. Other research findings from Credit Suisse suggest that global wealth inequality has increased over recent years, and that overall wealth in the US has grown faster than incomes – a possible precursor to recession. (*The Guardian*, 14 Oct 2014)

## ENERGY

➢ The UN Secretary–General, Ban Ki–Moon, praised Nicaragua’s efforts to promote sustainable energy in a visit to the country’s Camilo Ortega Wind Park. The wind park represents almost 25% of Nicaragua’s wind power, and will reduce CO_2_ emissions by at least 100 000 tons annually. Mr Ki–Moon spoke about Nicaragua’s vast potential for renewable energy, noting that it has already partially met the UN’s *Sustainable Energy for All* targets. He outlined the importance of energy in changing lives, improving the quality of life and promoting human dignity, and the importance of clean energy in addressing climate change; and called upon Nicaragua to continue investing in renewable energy to help everyone live a life of dignity. (*UN*, 29 Jul 2014)

➢ Re–starting Japan’s nuclear facilities after the 2011 Fukushima nuclear crisis may be delayed until 2015, increasing pressure on energy prices. Kyushu Electric Power’s Sendai plant will probably be the first to re–start following tightened safety rules after the 2011 disaster, reducing the purchase of fossil fuels by US$ 1.9 billion. All of Japan’s nuclear reactors closed after the nuclear crisis at Fukushima, forcing companies to import gas and coal to run power stations, leading to losses of US$ 34 billion and a government bailout for Kyushu. Electricity prices have increased as a result, and Hokkaido Electric Power have requested permission to raise household electricity rates by 17%, and Kyushu may be forced to follow suit. (*Reuters*, 6 Aug 2014)

➢ Following measures to save natural gas, Ukrainians are resorting to “wash visits” by using family members’ and friends’ shower facilities, as well as school closures and DIY insulation to cope with energy shortages. Authorities are trying to stockpile supplies ahead of winter, after Russia, its main supplier, stopped shipments in June following the accumulation of Ukrainian debts of US$ 5.3 billion. Gas supplies are half their normal capacity – insufficient to cover Ukraine’s winter needs. Homes must be kept cooler, and there are warnings of power cuts. Energy supplies are also affected by road and rail damage in the coal mining areas of Donetsk and Luhansk during the recent conflict. (*Bloomberg*, 18 Sep 2014)

➢ New York’s major, Bill de Blasio, committed to an 80% reduction in the city’s greenhouse gas emissions by 2050. This follows on from his predecessor Michael Bloomberg, whose 2007 greener–city agenda set out reducing emissions by 30% below their 2005 levels by 2030. Mr de Blasio’s plan focuses on making the city’s buildings more energy–efficient via strict regulations on new buildings and retro–fitting existing buildings. This is important as buildings contribute nearly 75% of the city’s CO_2_ emissions. The plan will be extremely expensive to implement (although many changes could be self–financing). It has proved difficult to reduce emissions by top–down agreements, although the city is fortunate to have had mayors who have recognised the facts and obstacles on climate change, and taken action. (*New York Times*, 22 Sep 2014)

➢ Nigeria, Africa’s largest economy, relies heavily on expensive diesel generators to supplement its unreliable national power grid. Its energy–generating capacity compares poorly with India and China, and the World Bank estimates that power shortages decrease Africa’s GDP growth by 2% annually, and by 4% in Nigeria. However, across Africa there is a rush to invest in energy capacity, which is estimated to increase by over 50% by 2020. Some of this increase should come from coal–fired power stations, but there are moves towards cleaner, renewable energy. This expansion is driven by more private investment in renewable energy, and its rapidly falling costs. Africa has some of the world’s best potential sites for wind, solar and hydropower, and could jump from being an energy laggard to a world leader in renewables, with the right leadership. (*The Economist*, 27 Sep 2014)

## ENVIRONMENT

➢ Mary Robinson, former President of the Republic of Ireland, has been appointed as special envoy for climate change. In 2010, she set up “The Mary Robinson Foundation – Climate Justice” to campaign for justice for victims of climate change. There was some urgency in her appointment because of the forthcoming 2014 Climate Summit, hosted by the UN Secretary–General. “Our work on climate justice emphasises the urgency of action on climate change from a people’s perspective and I intend to take this approach in my new mandate as special envoy for climate change,” said Mary Robinson. (*BBC*, 14 Jul 2014)

➢ The first global conference on health and climate change took place in Geneva, Switzerland. Climate change is already responsible for thousands of additional deaths each year, as rising temperatures cause diseases to spread to new areas, shifting weather patterns affect crops yields, and extreme events such as heat waves and floods degrade water supplies. Children and poor people are mainly affected by climate–related disease, and Dr Flavia Bustro, the WHO assistant director general of family, women’s and children’s health warns that “without effective action to mitigate and adapt to the adverse affects of climate change on health, society will face one of its most serious health challenges.” (*RTCC*, 27 Aug 2014)

➢ According to the UN’s World Meteorological Association (WMO), levels of atmospheric carbon dioxide (CO_2_) grew at the fastest rate for 30 years; with CO_2_ emissions being the biggest driver of global warming. WMO say that average daily levels reached their highest recorded level of 400 ppm (ppm) in May 2013, and some scientists warn that 350 ppm is the safe upper limit. The increase could be linked to faltering storage of carbon in the Earth’s oceans and forests, which currently locks away almost 50% of CO_2_ emissions, albeit at the cost of increased acidification. If this mechanism fails, the chances of avoiding dangerous climate change may be much reduced. The warnings come ahead of a UN climate summit in New York, aimed at bolstering efforts to reduce the burning of fossil fuels and resulting CO_2_ emissions. (*Financial Times*, 9 Sep 2014)

➢ At a Hong Kong conference, 11 Nobel laureates will unite to warn that humankind is living beyond its means, and darkening its future. They argue that the list of planetary ailments – global warming, deforestation, ocean acidification etc. – is lengthening, and that everyone must consider the environmental impact of each decision made. They urge more focus on cleaner energy sources, adaption of sustainability in wider contexts – food, water, soil and atmosphere – and for everyone to understand the benefits to humanity of adaptation to the dangers posed by over–exploitation of resources. (*The Guardian*, 7 Oct 2014)

➢ High consumption in developing countries combined with industrialisation in emerging economies have caused huge demand for raw materials, and the quantity of materials extracted for consumption increased by 60% in the past 30 years. 20% – more than 12 billion tonnes annually – ends up as waste. The global economy is set to grow by 400% by 2050 alongside rising populations, leading to questions of sustainability. One solution is to break the link between economic growth and materials use, by re–using and recycling products as much as possible. Product design can support this by eg, making products easy to disassemble and using recycled materials. This reduces input costs, making it cost–effective for businesses, plus being environmentally–beneficial. Consumer awareness on the importance of recycling and re–use is also vital for reducing waste and preserving resources. (*OECD*, 29 Oct 2014)

## FOOD, WATER AND SANITATION

➢ The WHO/UNICEF report *Progress on Drinking Water and Sanitation: 2014 update* shows that almost 2 billion people have gained access to improved sanitation and 2.3 billion to improved drinking–water since 1990, and overall the access gap between rural and urban areas is narrowing, although urban areas are generally better supplied. However, 748 million people still use unimproved drinking–water. Stark inequalities persist, with the majority of those without improved sanitation are poorer people in rural areas. There can also be striking inequalities in urban access, with people living in informal or illegal settlements less likely to have improved water or sanitation. “If we hope to see children healthier and better educated, there must be more equitable and fairer access to improved water and sanitation”, says Sanjay Wijesekera, UNICEF Chief of Water, Sanitation and Hygiene. (*WHO*, 8 May 2014)

➢ The Economist Intelligence Unit published a report on food loss and its intersection with food insecurity. It noted that globally there is enough food to feed everyone, but 1–in–8 people are chronically undernourished, with food waste and loss at the centre of the gap between production and consumption. It estimates that food loss amounts to US$ 750 billion each year. Food loss is correlated with food security and income level. For developing countries, it calls for improved farming methods, infrastructure and operating environments to increase efficiency and mitigate loss. The problem of food waste in developed countries could be addressed by eg, by food waste becoming socially unacceptable, and clarifying date labelling on food. Overall, the report calls for a more efficient and responsive global food supply chain, where currently–wasted surpluses in the developed world can be diverted to countries where supply is short. (*EIU*, 28 May 2014)

➢ The Millennium Development Goal for sanitation – halving the number of people without sustainable access to basic sanitation – is unlikely to be met, and according to the UN, 2.5 billion people still lack “improved sanitation facilities”. This is a mere 7% reduction from 1990, when 2.7 billion lacked access. This affects human dignity, health and the environment, and is cross–cutting – improved sanitation can reduce child mortality, improve maternal health and improve education, as better sanitation makes it easier for girls to attend school. The UN Deputy Secretary–General, Jan Eliasson, expects sanitation to figure highly in the post–2015 Sustainable Development Goals. “Invest in sanitation and you will have concrete results with positive changes for people’s lives,” he says, calling for more emphasis on urban areas with inadequate infrastructure. (*The Guardian*, 28 Aug 2014)

➢ Participants in the 2014 World Water Week emphasised the importance of a water goal, and the integration of energy and water in the post–2015 Development Agenda, and concluded that water efficiency is vital to fighting poverty and hunger. The event called for everyone to become as aware of water efficiency as they are of energy efficiency, for improved rainwater management to ensure development goals can be met, for the invention of a non–flush toilet to cut water usage, and for the need to grow biofuels in areas reliant on rainfall rather than irrigation. In addition, several prizes were awarded for water–related excellence, including to eThekwini Water and Sanitation (Durban, South Africa) for its approach to providing water and sanitation. (*World Water Week in Stockholm*, 5 Sep 2014)

➢ The UN report *The State of Food Insecurity in the World* shows that the numbers of hungry people has fallen sharply over the past 10 years, although 805 million – 1 in 9 of the global population are still under–nourished. The numbers of chronically under–nourished people fell by more than 100 million. The ambitious goal of halving the number of chronically under–nourished people by 2015 has been met by 25 developing countries, but will not be met globally, as success stories like Brazil are partially off–set by eg, Haiti, where numbers rose from 4.4 million in 1990–92 to 5.3 million in 2012–14. Ebola has also undermined food security in West Africa by endangering harvests and increasing food prices, and ongoing conflicts in Syria, South Sudan and the Central African Republic hinder humanitarian efforts to reach affected people. (*Reuters*, 16 Sep 2014)

## PEACE AND HUMAN RIGHTS

➢ The latest Global Peace Index (GPI) shows that nations spend an estimated US$ 9.8 trillion on dealing with violence, as peace deteriorated slightly for the seventh year, due to conflicts in Syria, South Sudan etc. The Institute for Economics and Peace (IEP), which produces the GPI, says that this is equivalent to 19% of global economic growth from 2012–13 – or US$ 1350 per person. The true cost may be much higher, as some data are impossible to obtain. Whilst major international conflicts are less common, internal conflicts are increasing. Eleven countries are in absolute conflict, but 500 million people live there, with 200 million living on less than US$ 2/d. The GPI lists Iceland, Denmark and Austria as the most peaceful nations, and identified Zambia, Haiti, Argentina, Chad, Bosnia and Herzegovina, Nepal, Burundi, Georgia, Liberia and Qatar as vulnerable to small to medium deteriorations in peace. (*The Guardian*, 8 Jun 2014)

➢ The African Court of Justice and Human Rights was intended to be an alternative to the International Criminal Court (ICC) in The Hague – criticised by African governments for targeting African leaders. However, the 2014 African Union decided to grant serving leaders and senior officials immunity from prosecution, unlike the ICC. Although only valid while people are in office, critics say that it could encourage attempts to seize power for life. This means that victims would have to turn to the ICC for justice rather than the African Court. The African court has gained support amongst some African people, as it dovetails with a culture of reconciliation, but Amnesty International describes it as “a step backward for justice”. (*The Guardian*, 3 Jul 2014)

➢ Data from the UNHCR shows that more than 3 million Syrians have fled from their country’s civil war – which has claimed 191 000 lives to date – with 1 million in the past year alone. This suggests increasingly horrific conditions within Syria, and places huge strains on their host countries, where nearly 40% of refugees are living in sub–standard conditions. The war has also displaced 6.5 million people internally, so almost 50% of Syrians have been forced to flee their homes. This has become the UNHCR’s largest relief operation, and it faces a funding shortfall of US$ 2 billion by this year alone. There are signs that the journey out of Syria is becoming more difficult, and the UNHRC is concerned about Syrians trapped inside the remote Iraqi al–Obaidi camp after agencies were forced to abandon the region after it was over–run by Islamic State jihadis. (*The Guardian/AFP*, 29 Aug 2014)

➢ According to a study by the Copenhagen Consensus Centre, the number of people killed in disputes between individuals globally – including domestic violence – is nine times higher than those killed in wars and conflicts, at an estimated cost of US$ 9.5 trillion a year. The authors note that physical violence in societies is a much larger problem than military violence, with graver economic consequences. In 2008, 1–in–3 countries had homicide rates of more than 10 per 100 000 – defined by the WHO as “epidemic”. It shows that 43% of all female homicide victims were killed by a current or former partner, 30% of women are subject to domestic violence, and 290 million children suffer violence in the home. “Domestic abuse of women and children should no longer be regarded as a private matter but a public health concern”, it says. (*The Guardian*, 9 Sep 2014)

➢ Indian children’s rights activist Kailash Satyarthi and Pakistani teenager Malala Yousafzai (its youngest recipient), who was shot by the Taliban for advocating girls’ rights to education, won the 2014 Nobel Peace Prize. They were chosen because of their struggle against the suppression of children, and for the right of all children to education. Kailash Satyarthi has headed various peaceful protests and demonstrations, focusing on the exploitation of children for financial gain. Malala Yousafzai was shot in northwest Pakistan in punishment for her campaigning blog against Taliban efforts to deny women an education. “The Nobel Committee regards it as an important point for a Hindu and a Muslim, an Indian and a Pakistani, to join a common struggle for education and against extremism,” said Thorbjoern Jagland, head of the Nobel Committee. (*Reuters*, 10 Oct 2014)

## SCIENCE AND TECHNOLOGY

➢ At the May 2014 *Big Data in Biomedicine* conference, Michele Barry (director of the Center for Innovation in Global Health at Stanford University) recorded a video interview, where she talked about the importance of big data for global health solutions. She outlined its importance for obtaining a clearer picture of population health, and would like to see big data surveillance used more widely in under–served areas, eg, sub–Saharan Africa, and amongst the world’s poorest billion people, who lack access to health care. She cited lack of funding and awareness as hampering efforts to use big data, and noted that cost–effective innovations, implemented pragmatically and preventatively, can support its usage. She said that big data can be used to identify dangerous pathogens for infectious disease control, which has positive outcomes for preventing of non–communicable diseases as they are often linked to infectious diseases. (*Scope*, 20 Jun 2014)

➢ The UN’s *Prototype Global Sustainable Development Report* says that crowd–sourcing could help to identify new development issues, and ensure that the global development agenda is more representative of views from the south. This follows on from a commitment at the Rio +20 summit to improve the science–policy interface and associated decision–making by bringing disparate scientific assessments into one place. It identified crowdsourcing as a way to gather opinions from more varied groups of scientists. Tracking progress will require significant capacity building of national statistics offices, and the use of technology such as remote sensing and big data analysis. However, a representative from the UK’s Overseas Development Institute doubts that the report will deliver much change, apart from monitoring global sustainable development, and calls for focused efforts at national levels to increase scientists’ impact. (*SciDev.Net*, 10 Jul 2014)

➢ Research published in *Nature* investigated the effects of malnutrition on healthy postnatal development of microbiota. Therapeutic food intervention saves lives in children with severe acute malnutrition, but incomplete growth restoration (“stunting”) is a major problem. The study found that severe malnutrition is associated with significant relative microbiota immaturity that is only partially offset by nutritional interventions. This suggests that longer food–based interventions and/or addition of gut microbes may be needed to achieve durable repair of children’s microbiota immaturity associated with malnutrition to improve clinical outcomes. (*Nature*, 19 June 2014)

➢ The 2014 Nobel Prize for Physiology or Medicine was awarded to John O’Keefe, May–Brit Moser and Edvard Moser for their work on the brain’s internal positioning system. John O’Keefe initially discovered this system in 1971, and his work shows that different sets of nerve cells were activated whenever a rat was in different locations, and argued that these cells form a map within the brain. Husband and wife team, May–Britt and Edvard Moser, discovered a different part of the brain that acts like a nautical chart, enabling the brain to judge distance and navigate. The Nobel committee said that “a better understanding of neural mechanisms underlying spatial memory is therefore important, and the discoveries of place and grid cells have been a major leap forward to advance this endeavour.” (*BBC News*, 6 Oct 2014)

➢ Researchers have grown human brain cells in a gel, where they formed networks akin to those in an actual brain. When the neurons were given the genes for Alzheimer disease, they formed the characteristic plaques and tangles. This could potentially accelerate the testing of new drugs to treat the condition. The technique could also be used to study the effects of genes that predispose some–one to develop Alzheimer disease, and to develop models on how it develops. (*New York Times*, 12 October 2014)

